# The XRE-DUF397 Protein Pair, Scr1 and Scr2, Acts as a Strong Positive Regulator of Antibiotic Production in *Streptomyces*

**DOI:** 10.3389/fmicb.2018.02791

**Published:** 2018-11-16

**Authors:** Ramón I. Santamaría, Laura Sevillano, Jesús Martín, Olga Genilloud, Ignacio González, Margarita Díaz

**Affiliations:** ^1^Instituto de Biología Funcional y Genómica, Departamento de Microbiología y Genética, Consejo Superior de Investigaciones Científicas, Universidad de Salamanca, Salamanca, Spain; ^2^Fundación MEDINA, Centro de Excelencia en Investigación de Medicamentos Innovadores en Andalucía, Granada, Spain

**Keywords:** *Streptomyces*, positive regulator, antibiotic production, xenobiotic response element, toxin–antitoxin

## Abstract

The xenobiotic response element (XRE) transcription factors belong to a regulator family frequently found in *Streptomyces* that are often followed by small proteins with a DUF397 domain. In fact, the pair XRE-DUF397 has been proposed to comprise toxin–antitoxin (TA) type II systems. In this work, we demonstrate that one of these putative TA-systems, encoded by the genes *SCO4441* and *SCO4442* of *Streptomyces coelicolor*, and denominated Scr1/Scr2 (which stands for **S**. **c***oelicolor* **r***egulator*), does not behave as a toxin–antitoxin system under the conditions used as was originally expected. Instead the pair Scr1/Scr2 acts as a strong positive regulator of endogenous antibiotic production in *S. coelicolor*. The analysis of the 19 *Streptomyces* strains tested determined that overexpression of the pair Scr1/Scr2 drastically induces the production of antibiotics not only in *S. coelicolor*, but also in *Streptomyces lividans*, *Streptomyces peucetius, Streptomyces steffisburgensis* and *Streptomyces* sp. CA-240608. Our work also shows that Scr1 needs Scr2 to exert positive regulation on antibiotic production.

## Introduction

*Streptomyces* are Gram-positive bacteria with a complex life cycle that includes the formation of mycelia and spores. In order to compete with other inhabitants of their ecosystems (mainly the soil), these bacteria have developed the capacity to produce a high number of extracellular hydrolytic enzymes and, also, secondary metabolites with antibiotic and antifungal activities among others (Chater, 2016). The production of these metabolites is tightly regulated through a large number of signal transduction proteins, including transcriptional regulators, which confer *Streptomyces* with the ability to rapidly respond to environmental changes by using available nutrients and producing secondary metabolites. It has been determined that 804 out of the 8300 genes in the genome of *Streptomyces coelicolor* are associated with this function. Of these, 499 have been classified as transcriptional regulators, 155 as one-component systems, 64 as sigma factors and 9 as DNA-binding proteins^1^ (Ortet et al., 2012). The xenobiotic response element (XRE) family of transcription factors (TF) is comprised of 70 TFs in *S. coelicolor.* This XRE family is the second most frequently occurring regulator family in bacteria, which control several diverse metabolic functions (Novichkov et al., 2013)^2^. Although these TF are abundant in *Streptomyces* genomes they have been poorly characterized. The most studied member of this group is the master regulator BldD from *S. coelicolor.* BldD is a small (18 kDa) protein that is a transcriptional regulator essential for morphological development and antibiotic production (den Hengst et al., 2010). WhiJ (SCO4543) is another member that has been studied in this organism, which has been associated with the repression of differentiation (Aínsa et al., 2010). WhiJ has a wide number of uncharacterized paralogous genes that are normally clustered with two additional genes. One of which, in the case of WhiJ, is SCO4542, a small protein belonging to the DNA-binding family that contains a domain of unknown function. This domain has been denominated DUF397 and is thought to interact with WhiJ, preventing it from binding to the operator sequence present in developmental genes (Aínsa et al., 2010). Actually, the DUF397-XRE gene pair encodes proteins that are most abundant in Actinobacteria, which have been assigned the function of class II toxin–antitoxin systems (TAS: TA-systems) among other functions (Makarova et al., 2009). In *S. coelicolor*, the XRE protein has been predicted to act as an antitoxin, with the associated small DNA-binding protein containing the DUF397 domain acting as a toxin; although its molecular mode of action has not yet been described (Sevin and Barloy-Hubler, 2007; Makarova et al., 2009; Doroghazi and Buckley, 2014).

Toxin–Antitoxin loci systems (TASs) are small genetic elements composed of a stable toxin and its cognate unstable antitoxin. The toxin, when released, prevents or alters cellular processes including translation, DNA replication, and ATP and cell wall synthesis and this activity can lead to cell death or the formation of drug-tolerant persister cells (Schuster and Bertram, 2013). Based on the molecular nature of the antitoxin modules, the TASs are currently grouped into six classes depending on their antitoxin nature: type II, IV, V, and VI are proteins and those in classes I and III are small regulatory RNAs (Lobato-Márquez et al., 2016). Type II class proteins are the most abundant and best described and include both the toxin and antitoxin, small proteins that form a stable complex. The antitoxin blocks the activity of the toxin by hiding the region responsible for toxicity (Goeders and Van Melderen, 2014; Hayes and Kedzierska, 2014).

The use of the TA finder 2.0 http://202.120.12.133/TAfinder/index.php (a TA-systems database web tool) predicted the presence of 42 TAS pairs in the *S. coelicolor* genome^3^, of which 15 are classified as XRE/DUF397 (Shao et al., 2011; Xie et al., 2018). In the present work, the putative TAS functionality of one of these XRE/DUF397 protein pairs from *S. coelicolor*, encoded by *SCO4441/SCO4442* and paralogous to *whiJ* and its downstream gene (*SCO4543/SCO4542*, respectively), was studied. Consequently it was found that the overexpression of the putative toxin SCO4442 was neither deleterious in the *S. coelicolor* wild-type strain or in the deletion mutant obtained in this work. These same results were obtained when *Streptomyces lividans* wild-type strain was used as the host. Therefore, this gene pair does not function as a toxin–antitoxin system, at least under the conditions assayed, as was originally predicted using bioinformatics.

Additionally, we found that the proteins encoded by SCO4441/4442 act as a positive regulator of endogenous antibiotic production in *S. coelicolor* and were named Scr1 and Scr2, respectively. The overexpression of Scr1, in combination with Scr2, drastically induces the production of antibiotics not only in *S. coelicolor*, but also in *S. lividans, Streptomyces peucetius*, *Streptomyces steffisburgensis*, and *Streptomyces* sp. CA-240608, as determined from the 19 strains tested. Analysis of the chromatographic peaks of the molecules induced in each case was performed, and an increment in some endogenous compounds and the appearance of new induced metabolites were detected. In conclusion, this protein pair seems to function as a positive regulator in the complex regulatory network of antibiotic production. These results open new doors to the application of Scr1/Scr2 in biotechnology, with the possibility of discovering new and natural products.

## Materials and Methods

### Strains, Media, and Growth Conditions

*Streptomyces* strains used in this study are: *S. albus* J1074, *S. argillaceus* ATCC 12596, *S. coelicolor* M145, *S. glaucescens* Tü49, *S. griseus* ATCC13273, *S. lividans* 1326, *S. parvulus* JI2283, *S. peucetius* ATCC 27952, *S. rochei* CECT 3329, *S. steffisburgensis* NRRL3193, *S. vinaceus* JI2838, and 8 *Streptomyces* sp. strains isolated from different soil samples (Supplementary Table S1). These strains were grown on R2YE, MS, PGA, and NA solid media for transformation, sporulation, conjugation, and phenotypic assays, respectively (Coco et al., 1991; Kieser et al., 2000). YES xylose (Sevillano et al., 2016) or NMMP (Kieser et al., 2000) containing 1% of xylose were used in the overexpression assays. Routine plasmid construction and plasmid isolation was done in *Escherichia coli* DH5α, and *E. coli* ET12567, a non-methylating strain, was used to obtain the plasmids to be transformed into *S. coelicolor*. *E. coli* strain BW25113 (pIJ790) (containing the λ Red system) (Datsenko and Wanner, 2000) and ET12567 (pUZ8002) (harboring the *tra* genes in the non-transmissible RP4-derivative plasmid pUZ8002) (MacNeil et al., 1992) were used for PCR-targeted mutagenesis of *S. coelicolor* M145 and conjugation plasmid transfer to the different *Streptomyces* species. *Staphylococcus aureus* MB5393, *E. coli* ATCC25922 and *Candida albicans* ATCC64124 were used in the antibiogram analysis. Antibiotics were used when needed for plasmid selection (*E. coli*: 100 μg mL^-1^ for ampicillin; 50 μg mL^-1^ for apramycin; 50 μg mL^-1^ for kanamycin; 34 μg mL^-1^ for chloramphenicol, and 25 μg mL^-1^ for nalidixic acid. *S. coelicolor*: 20 μg mL^-1^ for neomycin, 10 μg mL^-1^ for apramycin and 10 μg mL^-1^ for thiostrepton).

### DNA Manipulation

DNA transformation and manipulation of *E. coli* and *S. coelicolor* were carried out using the methods by Green and Sambrook (2012) and Kieser et al. (2000), respectively. The plasmids used in this work are listed in Table 1.

**Table 1:** Plasmids used.

Plasmid	Characteristics	Reference
pIJ773	pBluescript SK derivative conjugative plasmid containing the Apramycin resistance cassette.	Gust et al., 2003
pXHis1	pBluescript SK derivative. Ampicillin resistance. The *xysA* promoter from *S. halstedii* controls *xys1Δ* expression.	Adham et al., 2001a
pN702Gem3	High-copy number *E. coli*/*Streptomyces* shuttle vector. Neomycin resistance.	Fernández-Ábalos et al., 2003
pN702Gem3c	Conjugative pN702Gem3 derivative. A BamHI fragment of 1380 KB, from pIJ773, containing the oriT and the apramycin resistance was cloned in the BlgII.	This work
pNX4441	pN702Gem3 derivative. The *xysA* promoter from *S. halstedii* controls *SCO4441* expression. The protein SCO4441(Scr1) has His Tag at the carboxy terminal.	This work
pNX4442	pN702Gem3 derivative. The *xysA* promoter from *S. halstedii* controls *SCO4442* expression. The protein SCO4442 (Scr2) has His Tag at the carboxy terminal.	This work
pNX4441/42	pN702Gem3 derivative. The *xysA* promoter from *S. halstedii* controls *SCO4441* expression and the *SCO4442* is expressed under its own promoter control. The protein SCO4442 (Scr2) has His Tag at the carboxy terminal.	This work
pNX4441/42c	Conjugative pN702Gem3 derivative. A BamHI fragment of 1.38 kb, from pIJ773, containing the oriT and the apramycin resistance was cloned in the BlgII site of the plasmids pNX4441/42.	This work
pHJL401	Low-copy number *E. coli*/*Streptomyces* shuttle vector. Ampicillin and thiostrepton resistances.	Larson and Hershberger, 1986
pHJL401c	Conjugative pHJL401 derivative obtained by insertion of a HindIII-NheI band of 1,6 Kb from pN702Gem3c in the plasmid pHJL401 digested with HindIII-XbaI.	This work
pHAX41/42 c	Conjugative pHJL401 derivative. Obtained by insertion of a HindIII-NheI band of 3.8 kb from pNX4441/42 conj in the plasmid pHJL401 digested with HindIII-XbaI.	This work


### Mutant Generation

The coding regions of *SCO4441, SCO4442* or both genes were replaced by an apramycin resistance cassette (*aac(3)IV* gene) by using REDIRECT PCR-targeting technology (Gust et al., 2003). The primers LS-090 and LS-091 (Supplementary Table S2) were used to generate the mutation cassette from the plasmid pIJ773 (Gust et al., 2003), which was used as the template. The mutated genes were obtained using cosmid SCD6 (Redenbach et al., 1996) and transferred by conjugation from ET12567 (pUZ8002) to *S. coelicolor* M145. The desired mutants were selected by apramycin resistance and sensitivity to kanamycin. PCR assays confirmed the deletion of the *SCO4441, SCO4442* or both genes in *S. coelicolor* M145.

### Antibiotic Determination

Qualitative actinorhodin (ACT) production of the *S. coelicolor* strains was observed on different solid media. Approximately 10^5^ spores were deposited in 5 μL drops onto plates that were incubated at 30°C. ACT production was detected after three to 10 days of growth as a blue halo around the colonies.

Colorimetric quantification of prodiginines (RED) and ACT production was determined by the spectrophotometric method described in Yepes et al. (2011). All experiments were performed in triplicate.

### LC-HRMS-Analyses

Liquid cultures (10 mL) of *S. coelicolor* or *S. lividans* in YES+Xylose, or NMMP+Xylose, containing 20 μg mL^-1^ for neomycin or 10 μg mL^-1^ for apramycin, depending on the plasmid used, were incubated at 28°C for 8 days at 200 rpm. Then, 1 mL of the culture was extracted using 0.7 volume of 1% formic acid acidified ethyl acetate and the organic layer was dried *in vacuo*. The dry extracts were finally resuspended in methanol (100 μL). LC-HRMS-analyses were performed as previously described using a Bruker maXis QTOF mass spectrometer coupled to an Agilent 1200 LC (Martin et al., 2014; Perez-Victoria et al., 2016). Differential peaks where selected by direct comparison of the DAD signal base peak chromatograms.

### Plasmid Construction

The multicopy overexpression vectors pNX4441, pNX4442, and pNX4441-42 were derivatives of the pN703GEM3 plasmid (Fernández-Ábalos et al., 2003) and carry the neomycin resistance gene as selective marker. They were obtained by cloning the corresponding coding sequences *SCO4441* (*scr1*), *SCO4442* (*scr2*) or both, previously amplified by PCR using the corresponding oligonucleotides (see Supplementary Table S1), into the NdeI and XhoI sites of the intermediate plasmid pXHis1 (Adham et al., 2001b), yielding pX4441His, pX4442His, and pX4441/42His, respectively. In a second step, the corresponding BgIII/HindIII fragments of these three plasmids were cloned into the same sites of pN702GEM3, obtaining pNX4441, pNX4442, and pNX4441-42. In these constructs, the strong xylanase promoter *xysAp* (Rodríguez et al., 2005) controls the ORFs of *scr1, scr2* and *scr1* (*scr2* was under the control of its own promoter in pNX4441-42), respectively. In these plasmids the corresponding Scr1, Scr2, and Scr2 were tagged, respectively, with a six His tag at the carboxy terminus.

To obtain conjugative plasmids, a BamHI fragment of 1380 bp containing the *oriT* and the apramycin resistance, from pIJ773 (Gust et al., 2003), was cloned in the BlgII site of the plasmids pNX4441/42 and pN702Gem3, respectively, obtaining the plasmids pNX4441/42c and pN702Gem3c. Later a 3900-bp band was obtained from the plasmid pNX4441/42c and a 1600-bp band was obtained from the plasmid pN702Gem3c using the enzymes HindIII and NheI. These fragments were individually cloned into the low copy plasmid pHJL401 (Larson and Hershberger, 1986) digested with HindIII and XbaI to obtain the final plasmids pHAX4441/42c and its control pHJL401c, respectively. These plasmids carry resistance genes to thiostrepton and apramycin.

## Results

### Is Scr1/Scr2 a Functional TAS?

The *SCO4441* gene (*scr1*) encodes a 295 aa protein with a 7.18 isoelectric point, has a molecular weight of 33.59 kDa and has been predicted to be a putative antitoxin. This protein contains two regions, the HTH-XRE domain (aas 23 to 80) and the HipB domain (aas 24 to 62) (NCBI database^4^). The gene *SCO4442* (*scr2*) encodes a 63 aa protein with a 6.49 isoelectric point, has a molecular weight of 6.48 kDa and has been predicted to be a putative toxin. This protein contains a DUF397 region (aas 11 to 62).

As Scr1 and Scr2 are proposed to form part of a putative toxin–antitoxin system (TAS) (Shao et al., 2011; Xie et al., 2018), here we carried out a study to analyze the effect of its putative toxicity using the over-expression of Scr1, and/or Scr2 in *S. coelicolor* M145 and in *S. lividans* 1326. Three multicopy plasmids, pNX4441, pNX4442, and pNX4441/42 (see section “Materials and Methods”) were generated, where the expression of these genes was controlled by the xylanase strong promoter *xysAp* (Rodríguez et al., 2005). The plasmids and the control, the empty plasmid pN703Gem3, were introduced into both *Streptomyces* species to assess the possibility of Scr2 acting as a toxin.

**FIGURE 1:**
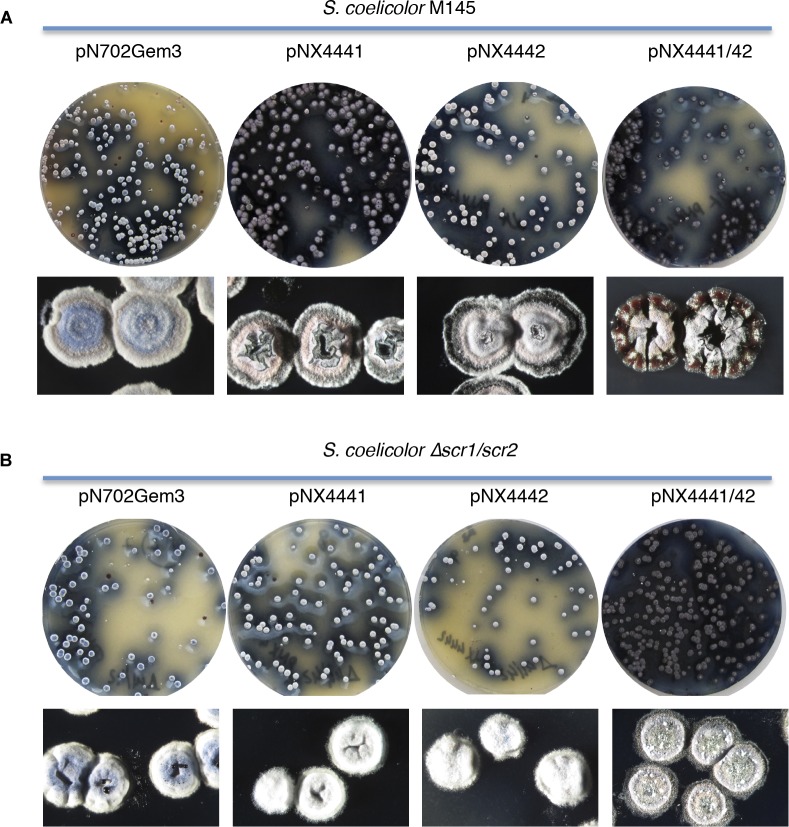
Overexpression of the putative SCO4441/SCO4442 TAS (Scr1/Scr2). **(A)** *S. coelicolor* M145 (wt); **(B)** *S. coelicolor Δscr1/scr2. In each panel:* colonies obtained after transformation with the different multicopy plasmids overexpressing the putative antitoxin Scr1 (pNX4441), the putative toxin Scr2 (pNX4442), both proteins Scr1/Scr2 (pNX4441/42) and the empty plasmid (pN702Gem3) in R2YE medium (Top); Detail of the morphology of the colonies obtained after 10 days (Bottom).

The *S. coelicolor* cells overexpressing the putative toxin in plasmid pNX4442 were viable, generating colonies which were able to differentiate like those obtained with both the control plasmid and the putative antitoxin (pNX4441). A slight delay in differentiation was observed when both genes of the putative TAS were overexpressed (pNX4441/42) (Figure 1A). The same results were obtained when the plasmids were transformed into *S. lividans* where no toxic effect was observed when the putative toxin encoded in the plasmid pNX4442 was expressed (data not shown).

These results indicated that the protein encoded by *scr2* was not acting as a conventional toxin, inducing lethality when overexpressed in *S. coelicolor* and *S. lividans*, at least under the conditions used (Figure 1A), as was the case of YefM/YoeBsl, isolated from *S. lividans*, which was previously characterized in our laboratory (Sevillano et al., 2012).

To exclude the possibility that a single gene copy of *scr1*, present in the *S. coelicolor* genome (wild-type), could be sufficient to counteract the toxicity of Scr2 overexpression, a deletion mutant strain lacking both putative TAS genes, Δ*SCO4441/42* (Δ*scr1/scr2*), was generated (see section “Materials and Methods”) and used as a host for plasmids pNX4441, pNX4442, and pNX4441/42. The results obtained when overexpressing these plasmids in the new mutant strain were similar to those described for the wild-type (Figure 1B). Hence, the function of these two genes did not correspond to a TAS under these particular conditions. Interestingly, the mutant Δ*scr1/scr2* did not show any phenotype divergent from that of the wild-type in the conditions used (see section “Discussion”).

### Involvement of Scr1/Scr2 in a Positive Regulation of Antibiotic Production in *S. coelicolor* and in *S. lividans*

**FIGURE 2:**
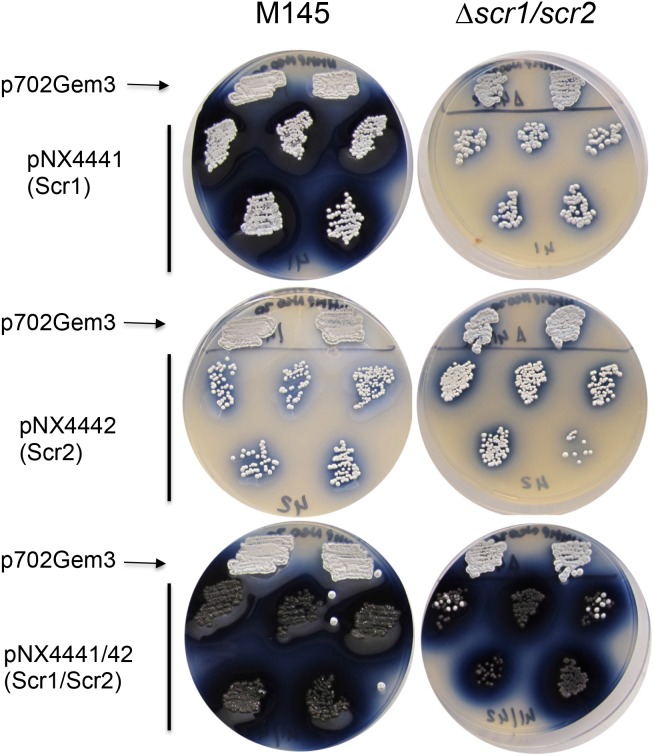
Study of the induction of ACT by overexpression of *scr1, scr2, and scr1/scr2 in S. coelicolor* M145 and *S. coelicolor Δscr1/scr2* strains on NMMP+xyl. Two clones are shown for the empty control, plasmid pN702Gem3, and five clones for those transformed with pNX4441 (Scr1), pNX4442(Scr2), or pNX4441/42 (Scr1/Scr2).

Furthermore, a clear phenotype of colored antibiotic induction was observed when the strains of *S. coelicolor* wild-type and Δ*scr1/scr2*, carrying the overexpression plasmid pNX4441/42, were grown on several solid media such as NMMP+Xyl. A high overproduction of the blue-red antibiotic ACT was observed when *scr1* was overexpressed either alone or with *scr2* in the wild-type strain. Nevertheless, when the *S. coelicolor* Δ*scr1/scr2* strain was used as a host, this high overproduction of ACT (blue color) was only observed with the overexpression of both genes at the same time (pNX4441/42) (Figure 2). These results indicated that Scr1 was a positive regulator of ACT production and required the presence of Scr2 to function.

To further delve into the functionality of these genes, two new mutants lacking only one of the two genes, *S. coelicolor Δscr1* and *S. coelicolor* Δ*scr2*, were generated (see section “Materials and Methods”). These two new single mutant strains, the double Δ*scr1/scr2* mutant and the wild-type were transformed with four plasmids, pNX4441, pNX4442, pNX4441/42, and pN702Gem3, and the production in liquid cultures was analyzed. The increase of the production of colored antibiotics was observed in all of the liquid media assayed [R2YE, NMMP+Xyl (data not shown) and YES+Xyl] when pNX4441/42 was used. Since the highest production was obtained in YES+Xyl, this medium was used to carry out the rest of the experiments.

**FIGURE 3:**
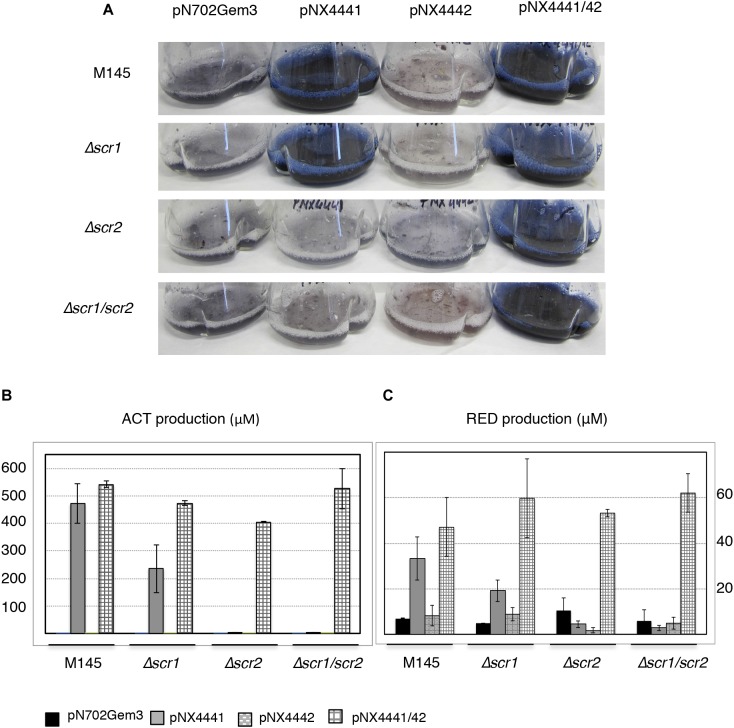
Actinorhodin (ACT) and RED induction by Scr1 and/or Scr2 in liquid YES+Xyl. **(A)** Colored antibiotic production induced by Scr1 (pNX4441), Scr2 (pNX4442), and Scr1/Scr2 (pNX4441/42) in different strains of *S. coelicolor* (M145, Δ*scr1*, Δ*scr2*, and Δ*scr1/scr2*); **(B)** colorimetric quantification of ACT production of the cultures shown in panel **A**; **(C)** colorimetric quantification of RED production of the cultures shown in panel **A**. The cultures were grown in YES+Xyl for 8 days. Error bars correspond to three independent experiments.

Once again, a high level of ACT was induced by Scr1 (pNX4441) in the wild-type strain. This induction was also observed in the *S. coelicolor Δscr1* strain, but not in the two strains lacking the *SCO4442* gene (Δ*scr2* and Δ*scr1/scr2*) (Figure 3A). Overproduction of the colored antibiotics was also observed in strains Δ*scr2* and Δ*scr1/scr2*, but only when the plasmid pNX4441/42 was used (Figures 3A,B). Antibiotic production in these cultures was quantified, and the yield of ACT was between 450 and 550 μM after expressing Scr1 alone or together with Scr2 in the wild-type M145 strain. The production of prodiginines (RED) was also induced under the same conditions reaching concentrations of 35–45 μM in this strain (Figure 3C). The quantification of these antibiotics in the Δ*scr2* and Δ*scr1/scr2* transformant strains reinforced the hypothesis that Scr1 acts as a positive regulator of antibiotic production and requires the presence of the protein encoded by *scr2*. Therefore, these two genes must act in conjunction (Figures 3B,C).

**FIGURE 4:**
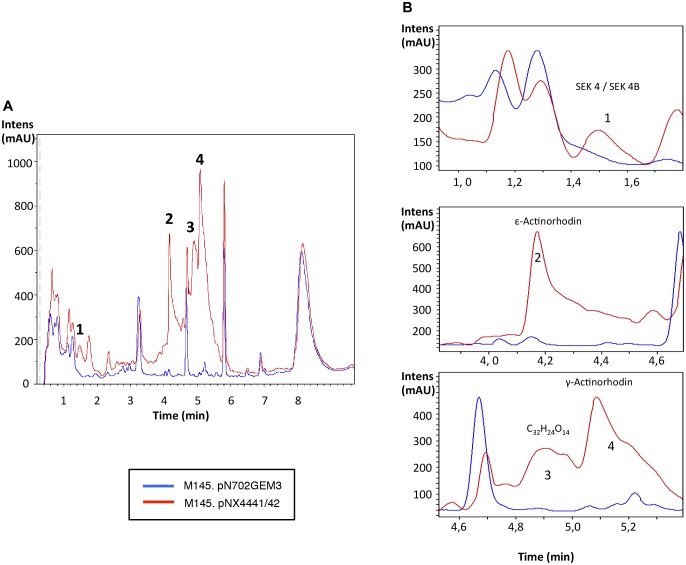
Metabolites induced by Scr1/2 in *S. coelicolor.* **(A)** Metabolite production induced by the overexpression of Scr1/Scr2 (pNX4441/42) in *S. coelicolor* M145 in YES+Xyl and detected by UV-Vis absorbance (200–900 nm) base peak chromatography. **(B)** Amplified region of the four most different peaks produced and their predicted compounds (1) SEK 4/SEK 4B; (2) ε-actinorhodin; (3) C_32_H_24_O_14_; (4) γ-actinorhodin.

Additionally, the analysis of the *S. coelicolor* M145 cultures overexpressing Scr1 and Scr2 by LC-HRMS was performed for identifying new putative compounds, originating from silent pathways that may be promoted by Scr1/Scr2. Several compounds were produced *de novo* by the action of Scr1/2 and were putatively identified as SEK 4, SEK 4b, ε-actinorhodin and γ-actinorhodin, and one putative new compound with the molecular formula C_32_H_24_O_14_ and an UV absorbance similar to actinorhodin (Figure 4). SEK 4 and SEK 4b have been previously described as shunt polyketide intermediates, non-enzymatically cyclized products from the biosynthesis of actinorhodin (Fu et al., 1994; Jetter et al., 2013).

**FIGURE 5:**
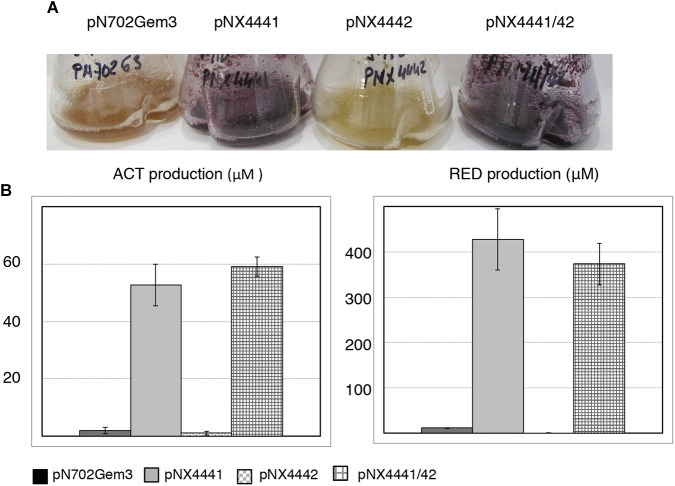
Antibiotic induction by Scr1, Scr2 and Scr1/Scr2 in *S. lividans* 1326 in liquid YES+Xyl. **(A)** Colored antibiotic production induced by Scr1 (pNX4441), Scr2 (pNX4442), and Scr1/Scr2 (pNX4441/42). **(B)** Colorimetric quantification of ACT and RED production of the cultures shown in panel **A**. Control: empty plasmid pN702Gem3. The cultures were grown in YES+Xyl for 8 days. Error bars correspond to three independent experiments.

**FIGURE 6:**
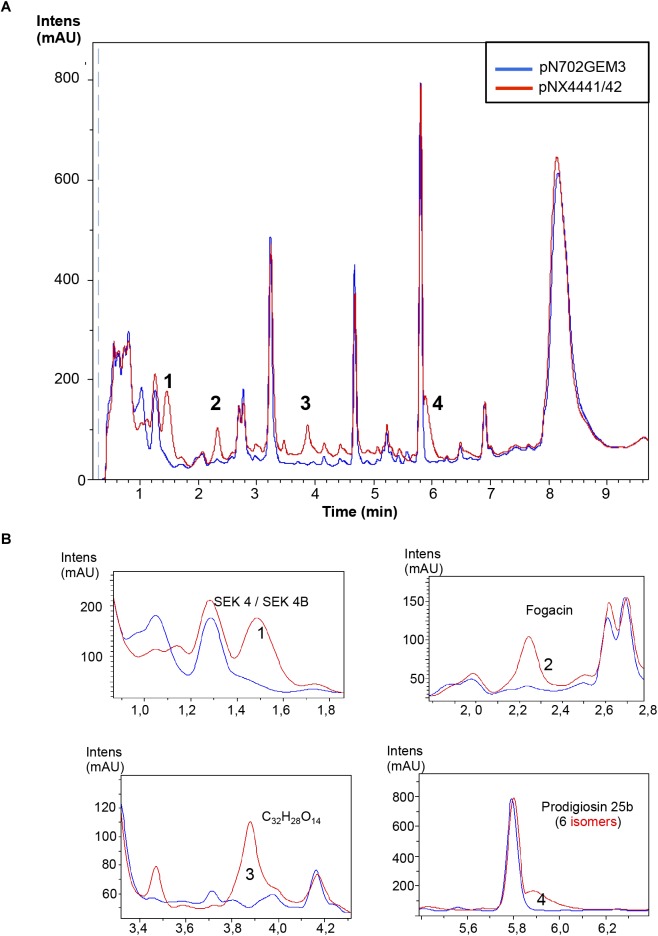
Metabolites induced by Scr1/2 in *S. lividans.* **(A)** Metabolite production induced by overexpression of Scr1/Scr2 (pNX4441/42) in *S. lividans* in YES+Xyl and detected by UV-Vis absorbance (200–900 nm) base peak chromatography. **(B)** Amplified region of the four most different peaks produced and their predicted compounds (1) SEK 4/SEK 4B; (2) Fogacin; (3) C_32_H_28_O_14_; (4) Prodigiosin 25b.

The ability of Scr1, Scr2, and Scr1/Scr2 from *S. coelicolor* to induce antibiotics was also tested in *S. lividans* 1326 in the same way as above. The plasmids pN702Gem3, pNX4441, pNX4442, and pNX4441/42 were introduced in this strain, cultured in liquid YES+Xyl and compared. As in *S. coelicolor*, production of ACT (59 μM) and RED (430 μM) was detected when this strain was transformed with the plasmids pNX4441 and pNX4441/42, but not observed when transformed with pNX4442. Interestingly the production of RED was almost ten times higher than the production reached for this antibiotic in *S. coelicolor* (Figures 5A,B). LC-HRMS analysis of the cultures of *S. lividans* 1326 overexpressing *scr1* and *scr2* detected the production of SEK 4, SEK 4b, fogacin, a putative new compound with the molecular formula C_32_H_28_O_14_ and a prodigiosin of the molecular formula C_25_H_33_N_3_O with six isomeric prodigiosins found in the Dictionary of Natural Products (DNP) (Figure 6). Fogacin has been previously described as a cyclic octaketide obtained from a *Streptomyces* sp. (strain Tü 6319) isolated from a contaminated soil in Romania (Radzom et al., 2006).

### Scr1/Scr2, a New Genetic Tool to Activate Secondary Metabolite Production in *Streptomyces* Species

**FIGURE 7:**
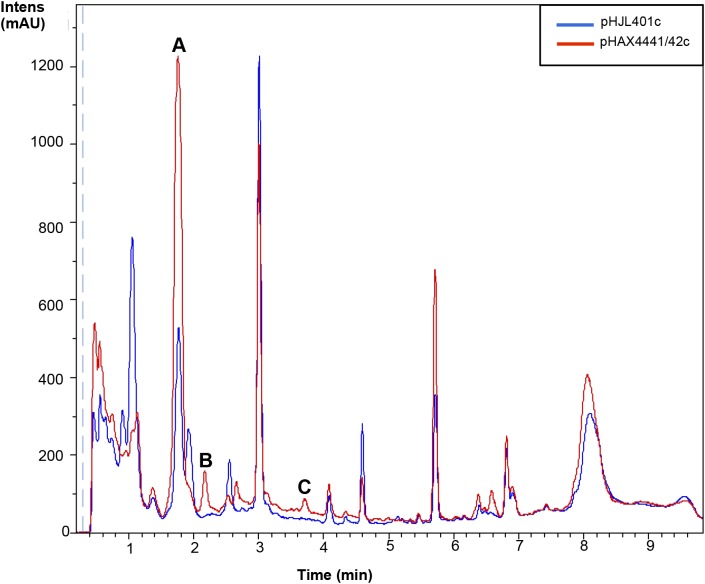
Metabolites induced by Scr1/2 in *S. peucetius.* Metabolite production induced by overexpression of Scr1/Scr2 (pHAX4441/42c) in YES+Xyl for 8 days and detected by UV-Vis absorbance (200–900 nm) base peak chromatography. Peaks A, B, C: unknown compounds.

**FIGURE 8:**
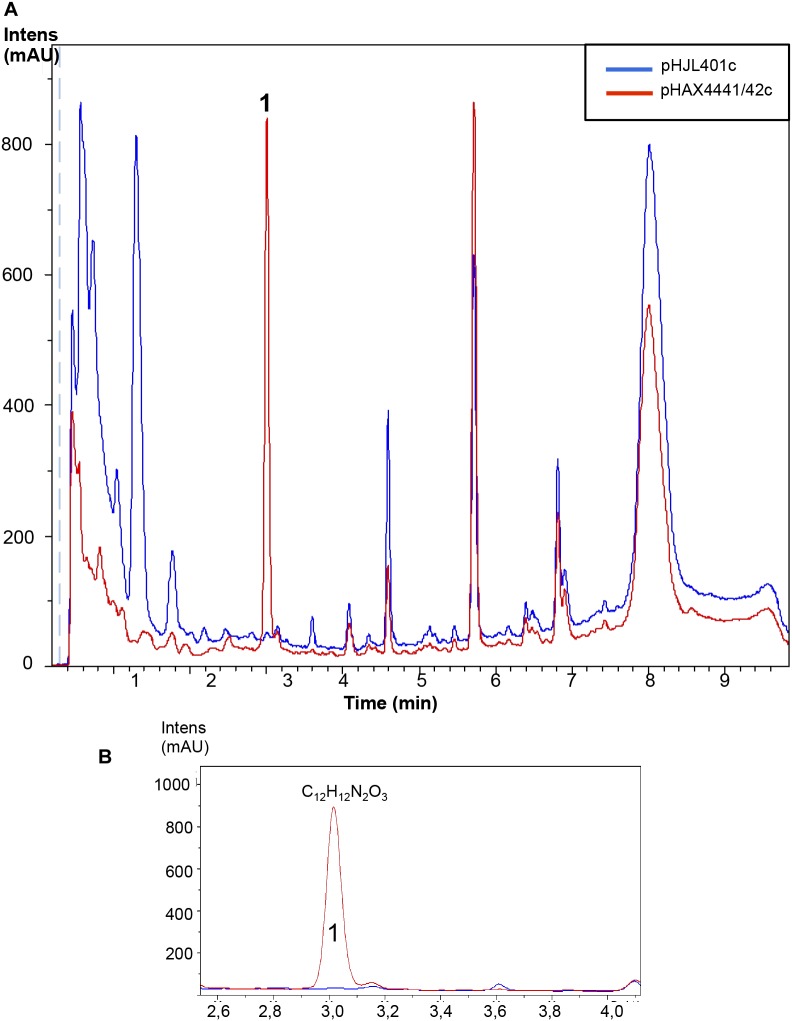
Metabolites induced by Scr1/2 in *S. steffisburgensis.* **(A)** Metabolite production induced by overexpression of Scr1/Scr2 (pHAX4441/42c) in *S. steffisburgensis* in YES+Xyl for 8 days and detected by UV-Vis absorbance (200–900 nm) base peak chromatography. **(B)** Amplified region of the peak produced and their predicted compound: N-[1-Hydroxy-2-(1H-indol-3-yl)-2-oxoethyl]acetamide.

A potential strategy for improving or inducing cryptic metabolites of different species of *Streptomyces* producers is the cloning of a positive regulator of antibiotic production. Based on the results obtained from the overexpression of Scr1/2 in *S. coelicolor* and *S. lividans*, these genes appeared to be good candidates for obtaining new natural products using this type of strategy. Therefore, we generated a plasmid that could be used to transfer these regulators into other *Streptomyces* species. To do so, a conjugative plasmid derived from pHJL401 was generated (pHJL401c) and used to introduce *scr1* and *scr2* in the final plasmid pHAX4441/42c. These two new plasmids were transferred from *E. coli* to nine *Streptomyces* species known to produce different antibiotics: *S. albus, S. argillaceus, S. glaucescens, S. griseus, S. parvulus, S. peucetius*, *S. rochei, S. steffisburgensis* and *S. vinaceus.* The conjugants obtained were grown in liquid YES+Xyl containing 10 μg mL^-1^ of apramycin for 8 days and the metabolites produced and extracted with acidified ethyl acetate were analyzed using LC-HRMS to look for the increased production of known or new compounds. This strategy was successful in *S. peucetius* and *S. steffisburgensis.* In *S. peucetius*, two putative new compounds were produced (peaks B and C) and an additional one (peak A) was greatly induced by Scr1/Scr2 (Figure 7). The analysis of the differentially detected peaks did not correspond with those of known compounds. This may have occurred in two of the cases (peaks A and B) due to the lack of ionization in both positive and negative ESI. In the case of peak C, the only component found in the DNP with the predicted molecular formula C_18_H_14_N_2_O_4_ (Supplementary Figure S1) corresponded to Flazine methyl ester, which had an UV spectrum that did not coincide with the spectrum determined experimentally (Buckingham, 2017). In *S. steffisburgensis*, an induced compound, N-[1-Hydroxy-2-(1H-indol-3-yl)-2-oxoethyl]acetamide, was highly produced and putatively identified (Figure 8). No changes were observed in the peaks obtained in the other studied strains (Supplementary Figures S2–S5).

**FIGURE 9:**
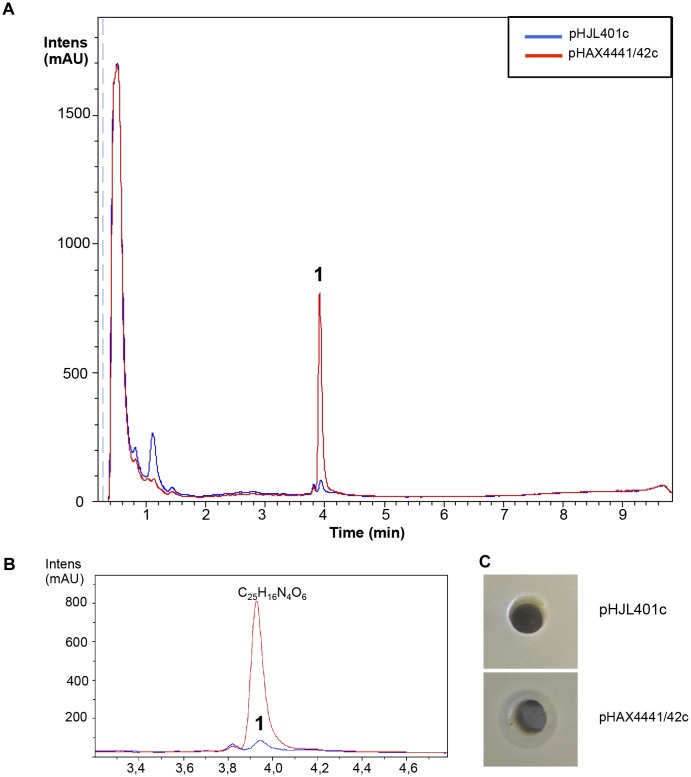
Metabolites induced by Scr1/2 in *Streptomyces* sp. *CA-240608.* **(A)** Metabolite production induced by overexpression of Scr1/Scr2 (pHAX4441/42c) in *Streptomyces* sp. CA-240608 in YES+Xyl and detected by UV-Vis absorbance (200–900 nm) base peak chromatography. **(B)** Amplified region of the peak produced and their predicted compound C_25_H_16_N_4_O_6_: Izumiphenazine A/B, Phenazinoline D/E. **(C)** Antibiogram against *E. coli* using 100 μL of the supernatant of *Streptomyces* sp. CA-240608 cultures carrying the indicated plasmid.

Moreover, eight additional strains of *Streptomyces* sp., with no clear antibiotic activity against the microorganisms tested (*S. aureus*, *E. coli*, and *C. albicans*) when grown in seven different types of media, were also used in the experiment. Conjugants were only obtained for two of the strains: *Streptomyces* sp. CA-240608 and *Streptomyces* sp. CA-258987. LC-HRMS-analysis of liquid culture extracts of *Streptomyces* sp. CA-240608 detected the overproduction of one component with the molecular formula of C_25_H_16_N_4_O_6_ (Supplementary Figure S1) that could correspond to any of the isomers Izumiphenazine A, Izumiphenazine B, Phenazinoline D or Phenazinoline E (Figure 9). These were the only compounds described in the DNP with this predicted molecular formula. Interestingly, the antibiogram activity of the clones of this strain overexpressing Scr1/Scr2 exhibited slight antibiotic activity against *E. coli*. By contrast, the control strain did not show any antibiotic activity (Figure 9). No changes in the production pattern of the strain *Streptomyces* sp. CA-258987 were detected (Supplementary Figure S5).

Therefore, these results validate the use of Scr1/Scr2 as a biotechnological tool for increasing or promoting different metabolite pathways in some *Streptomyces* species. The overexpression of Scr1/Scr2 strongly activates antibiotic production in *S. coelicolor* and *S. lividans*, and may be useful as a means for searching for new natural products in some *Streptomyces* species.

## Discussion

In this work, the positive role of the Scr1/Scr2 proteins in the regulation of antibiotic production has been established. This role has not been described previously and their function as a putative toxin–antitoxin system has only been inferred by bioinformatic comparison (Shao et al., 2011; Xie et al., 2018). One orthologous gene of *scr1*, with 99% identity, was previously cloned from *S. lividans* and denominated *cpb1*. The protein encoded for this gene was identified as a DNA binding protein that specifically binds to the promoter of the chitinase encoding gene *chiA*. Deletion of the gene *cpb1* partially relieves the glucose repression of chitinase production and chitinases are produced up to wild-type levels when grown in a medium containing colloidal chitin without glucose (Fujii et al., 2005). A qualitative experiment has been conducted, comparing the chitinase halo of the *S. coelicolor* wild-type strain and the Δ*scr1* in the presence of 0.15% colloidal chitin with or without glucose. No clear differences were observed for both strains (Supplementary Figure S6A).

Based on sequence homology, Aínsa et al. (2010) classified the gene *scr1* (*SCO4441*) as one of the 26 *whiJ* (*SCO4543*) paralogs on the *S. coelicolor* chromosome. These *whiJ* genes vary considerably with respect to their conservation in other organisms, being widespread in streptomycetes but generally absent from any other bacterial genomes. The *scr1* gene is present in all *Streptomycineae* and also in other complex actinomycetes such as *Kitasatospora, Microtetraspora, Streptosporangium* (Chandra and Chater, 2014). In *S. coelicolor*, most of these *whiJ* paralogs (22 out of 26 members) are neighbors of genes that encode either a very small binding protein with a DUF397 domain, like in the case of Scr2 (SCO4442), or by an antisigma factor or by both (Chandra and Chater, 2014). A model in which WhiJ binds to some operator sequences repressing developmental genes has been described. Interestingly, this repression might be released by its interaction with the small WhiJ-associated protein, SCO4542 (*scr2* paralog), whose activity is prevented by an unknown signal (Aínsa et al., 2010). These authors demonstrated that the total deletion of *whiJ* (*scr1* paralog) does not lead to a clear effect. However, the deletion of *SCO4542* causes colonies to appear bald (*bld*) in MSA medium and results in the *whi* phenotype in R2YE. Both of these phenotypes were suppressed by the simultaneous deletion of *whiJ*. The authors also proposed that the deletion of *SCO4542* would result in the constitutive binding of WhiJ to its target(s), repressing development and leading to a *bld* colony phenotype. In other words, SCO4542 prevents WhiJ from binding to target DNA and represses development (Aínsa et al., 2010).

Another protein containing a DUF397 conserved domain, which is critical for the correct progression of the developmental program and antibiotic production, is BldB (SCO5723) (Eccleston et al., 2006; Akpe San Román et al., 2010). It has been suggested that a partner, included in the Hpb (for helix-turn-helix partner of BldB) family, modulates its activity (Eccleston et al., 2006; Doroghazi and Buckley, 2014). Hpb is predicted to have an XRE-class helix-turn-helix domain, and diversification of the *bldb/hpb* family gene pairs suggests that a mutation in one gene encourages a compensatory change in its partner at the same locus. Based on different aspects of the relationship between this gene pair, some authors have proposed that the proteins containing the XRE domain function as antitoxins whereas DUF397 family proteins are novel toxins (Makarova et al., 2009; Doroghazi and Buckley, 2014).

As stated previously, *S. coelicolor* has 15 XRE-DUF397 pairs that have been predicted to act as toxin–antitoxin systems (Shao et al., 2011; Xie et al., 2018). Until now, this group of genes has not been studied experimentally. In this work, we chose to begin by studying the gene pair comprised of *scr1/scr2* in an attempt to describe the mode of action of a new group of TAS. However, under the conditions used in this work, this system did not behave as a typical TAS on which overexpression of the putative toxin (Scr2 in this case), in the absence of adequate levels of the putative antitoxin, (Scr1 in our study) produces a lethal effect. In our experiments, the number of colonies obtained in the transformation with the plasmids containing the antitoxin, the toxin or both genes was similar suggesting the no toxic effect of the toxin.

The mutation of these genes did not have a drastic phenotype when grown on several of the media assayed (Supplementary Figure S6B). Only the Δ*scr1* strain showed an accelerated differentiation when grown in NA solid media. No other clear differences were observed for the different strains in R2YE, R5A, or in MSA, as observed by Aínsa et al. (2010) with WhiJ/SCO4542 pair.

According to our results it is possible that Scr1 and Scr2 interact, although evidence of this interaction could not be obtained using purified proteins due to the insolubility of Scr2. Nevertheless, an indirect relationship was observed in *S. coelicolor* using strains Δ*scr2* and Δ*scr1/scr2* overexpressing Scr1. Induction of colored antibiotics was only observed when overexpression of Scr1 and Scr2 was carried out in these strains, but not with Scr1 on its own.

It is worth noting that several additional products of ACT and RED were induced in *S. coelicolor* by the overexpression of the regulator Scr1 (Figure 4). For example, production of the compounds Sek4 and Sek4b were detected in this strain. These two products were also detected in *S. lividans*. In this species, fogacin was also overproduced due to the overexpression of Scr1 and Scr2. This compound differs from all known intermediates of actinorhodin described and is a product of stereospecific cyclization and miscellaneous tailoring steps (Radzom et al., 2006). To our knowledge, the production of these three compounds has not been previously reported in *S. lividans*. In addition, the induction of other compounds was observed under the culture conditions used. This was the case for *S. steffisburgensis* in which the production of the putative N-[1-Hydroxy-2-(1H-indol-3-yl)-2-oxoethyl] acetamide was detected. This compound was previously described in *Streptomyces ramulosus* Tü 34 (Chen et al., 1983). Here, the overexpression of Scr1/Scr2 in a strain that did not present any clear antibiotic activity against Gram positive, Gram negative bacteria or yeasts, under different culture conditions, was also analyzed. The overexpression of Scr1/Scr2 in this strain permitted us to detect a weak antibiotic activity against *E. coli* that was absent in the control strain. Under these conditions, this strain, *Streptomyces* sp. CA-240608, produces izumiphenazines A/B or Phenazinoline D/E (Figure 9). The production of izumiphenazines A/B was previously reported from *Streptomyces* sp. IFM 11204, a strain isolated from a soil sample collected from Izumi forest in Chiba city, Japan. These compounds exert moderate activity over TRAIL-resistant AGS cells, a cell line typically used for evaluating cancer cell apoptosis (Abdelfattah et al., 2010). The compound Phenazinoline was previously reported from *S. diastaticus* YIM DT26, isolated from a soil sample collected in Yunnan, China (Ding et al., 2011), and from the strain of *S. fradiae* A196 that was isolated from hailstone in Gijón, Spain (Sarmiento-Vizcaino et al., 2018). These results suggest that this strategy may be useful for some *Streptomyces* strains for increasing the level of production of some compounds. However, the regulators analyzed do not present a general activating action in all of the strains studied, as was initially expected.

## Author Contributions

RS and LS conducted most of the experiments. JM performed the LC-HRMS-analysis. LS, JM, IG, OG, MD, and RS analyzed the results. MD, LS, and RS conceived the experiments and wrote the manuscript. All authors have read and approved the final manuscript.

## Conflict of Interest Statement

The authors declare that the research was conducted in the absence of any commercial or financial relationships that could be construed as a potential conflict of interest.

## Footnotes

1 http://www.p2tf.org

2 http://regprecise.lbl.gov/RegPrecise/collection_tffam.jsp?tffamily_id=70

3 http://202.120.12.133/TAfinder/report.php?job_id=XZiXKD6mm

4 https://www.ncbi.nlm.nih.gov/protein/NP_628608.1
