# Supplementary material for: The XRE-DUF397 Protein Pair, Scr1 and Scr2, Acts as a Strong Positive Regulator of Antibiotic Production in Streptomyces
Source: Front Microbiol. 2018 Nov 16;9:2791. doi: 10.3389/fmicb.2018.02791 (PMC6262351; doi:10.3389/fmicb.2018.02791)
Supplement: Supplementary file 1 [file Data_Sheet_1.PDF]

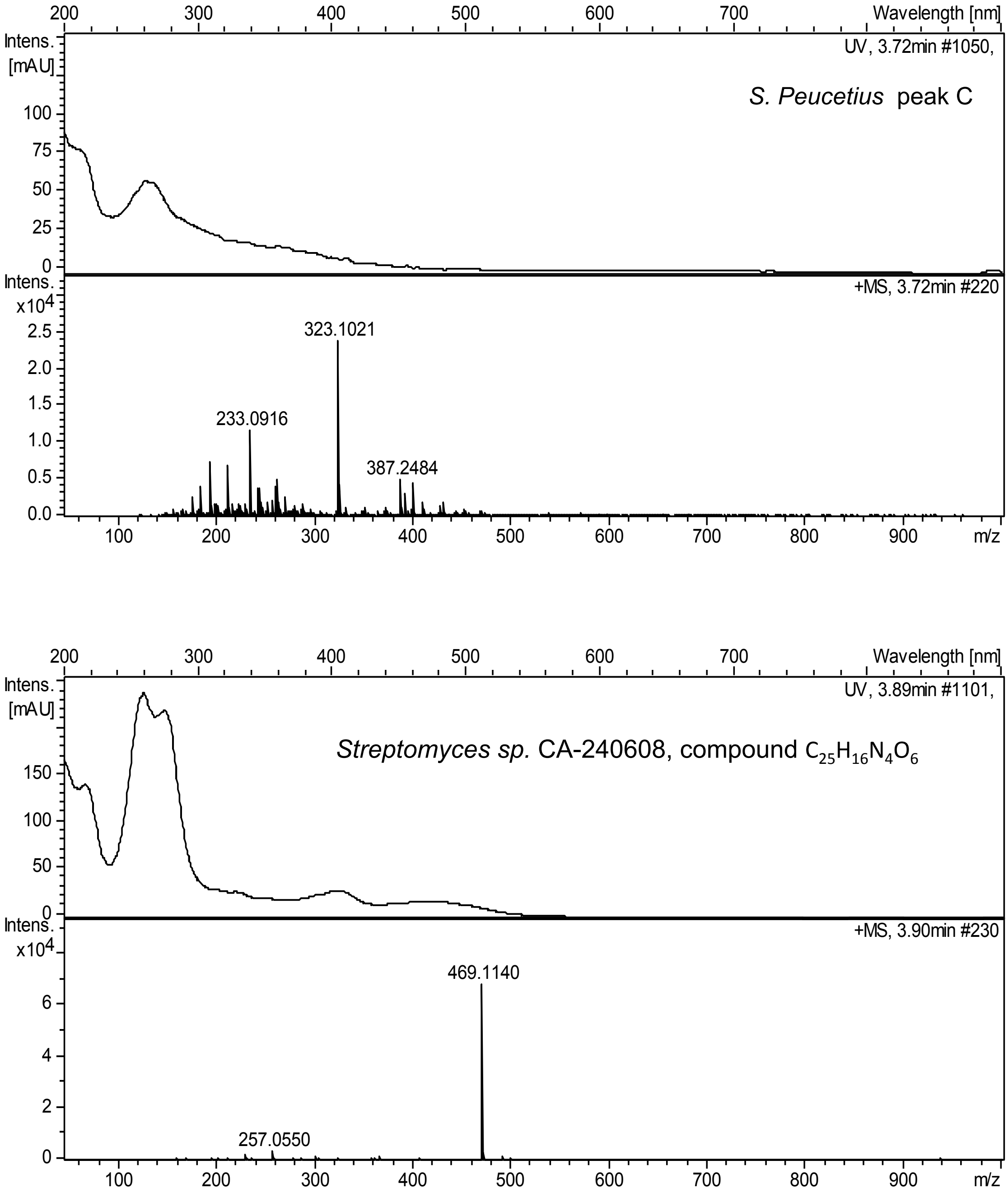

Figure S1: Mass spectrometry of peak C from *S. peucetius* ($C_{18}H_{14}N_2O_4+H^+=$ 323.102633. Experimental value m/z=323.1021. Error= -1.6ppm) and of the compound with formula $C_{25}H_{16}N_4O_6$ from *Streptomyces* sp. CA-240608 ($C_{25}H_{16}N_4O_6$+H+= 469.114261. Experimental value m/z=469.1140. Error= 0.6ppm) .
