# Supplementary figures and images for: The XRE-DUF397 Protein Pair, Scr1 and Scr2, Acts as a Strong Positive Regulator of Antibiotic Production in Streptomyces
Source: Front Microbiol. 2018 Nov 16;9:2791. doi: 10.3389/fmicb.2018.02791 (PMC6262351; doi:10.3389/fmicb.2018.02791)

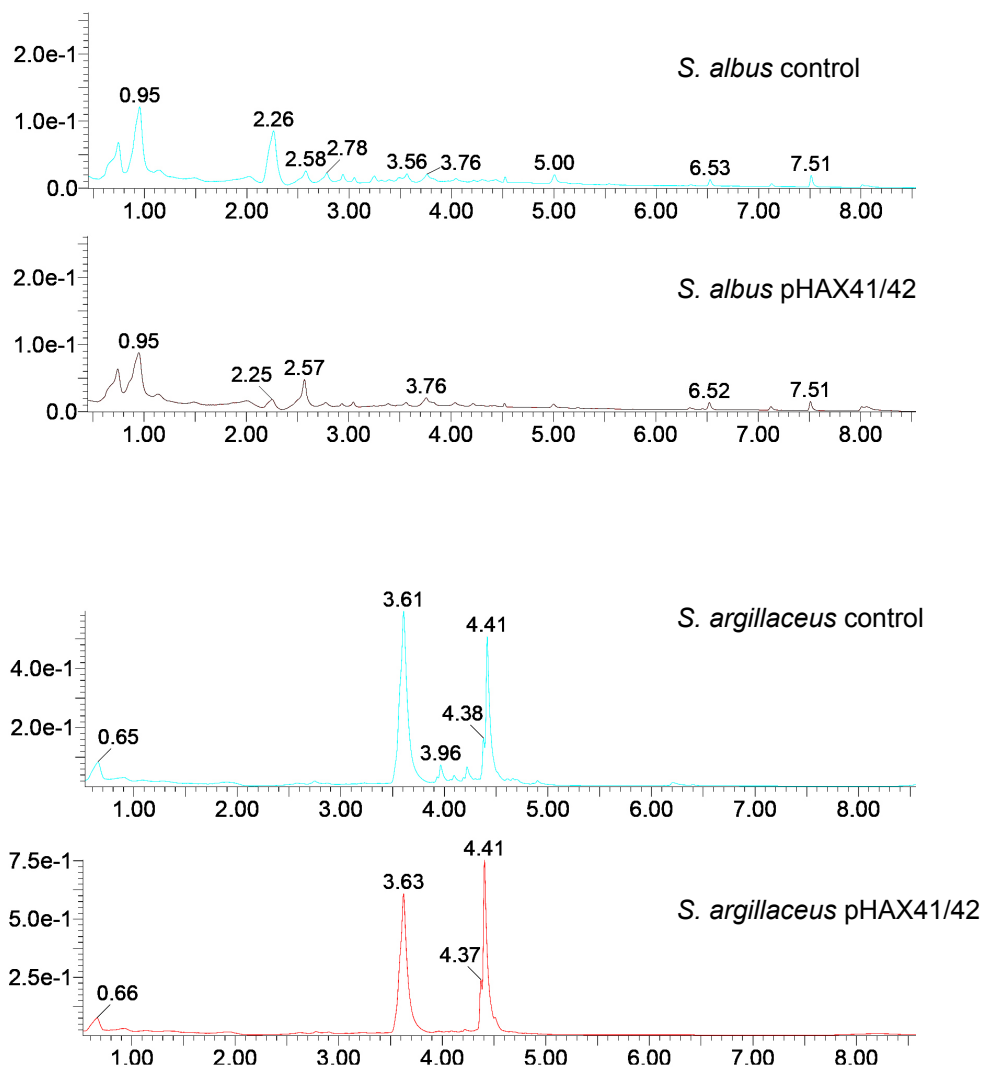

Figure S2: Chromatograms of *S. albus* and *S. argillaceus* grown in liquid YES+Xyl for 8 days.

Supplement: Supplementary file 2 [file Data_Sheet_2.PDF]

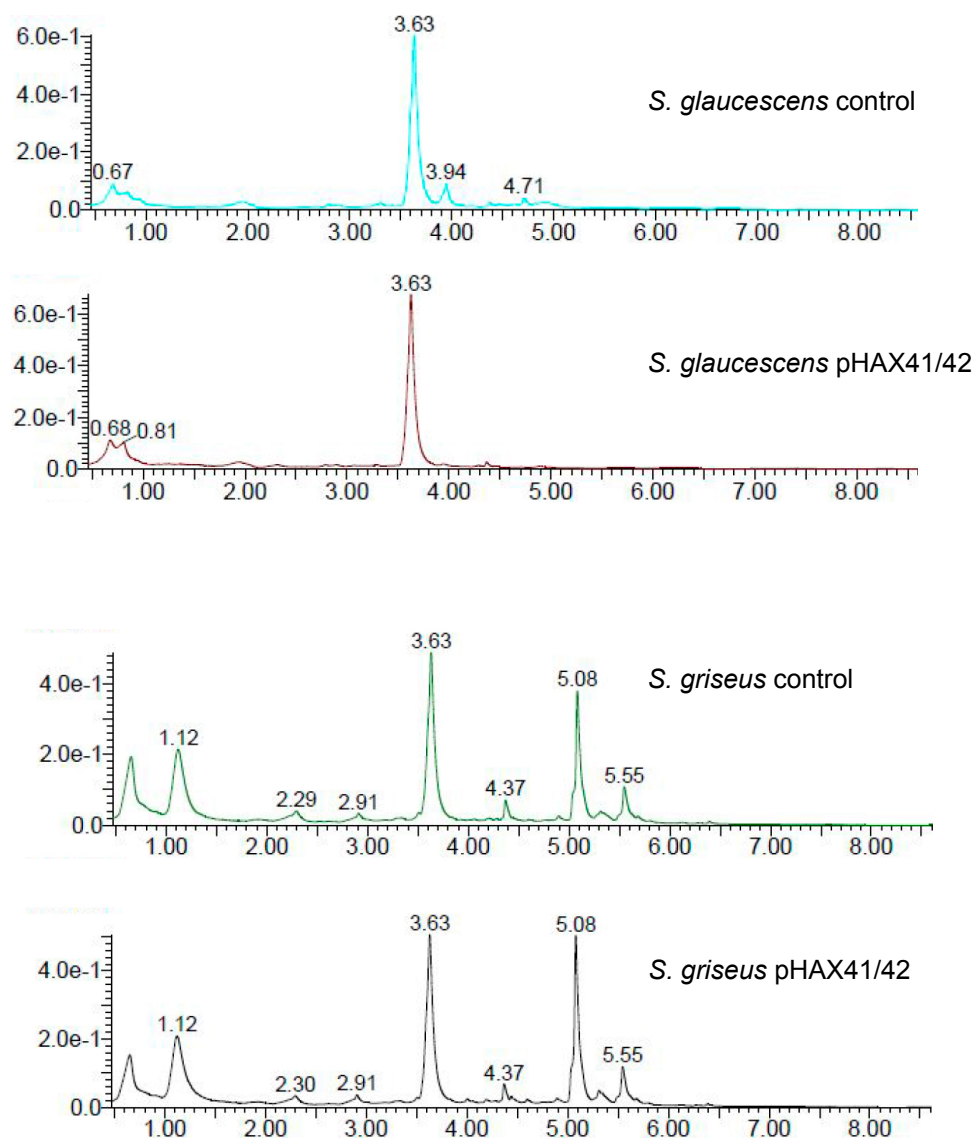

Figure S3: Chromatograms of *S. glaucescens* and *S. griseus* grown in liquid YES+Xyl for 8 days.

Supplement: Supplementary file 3 [file Data_Sheet_3.PDF]

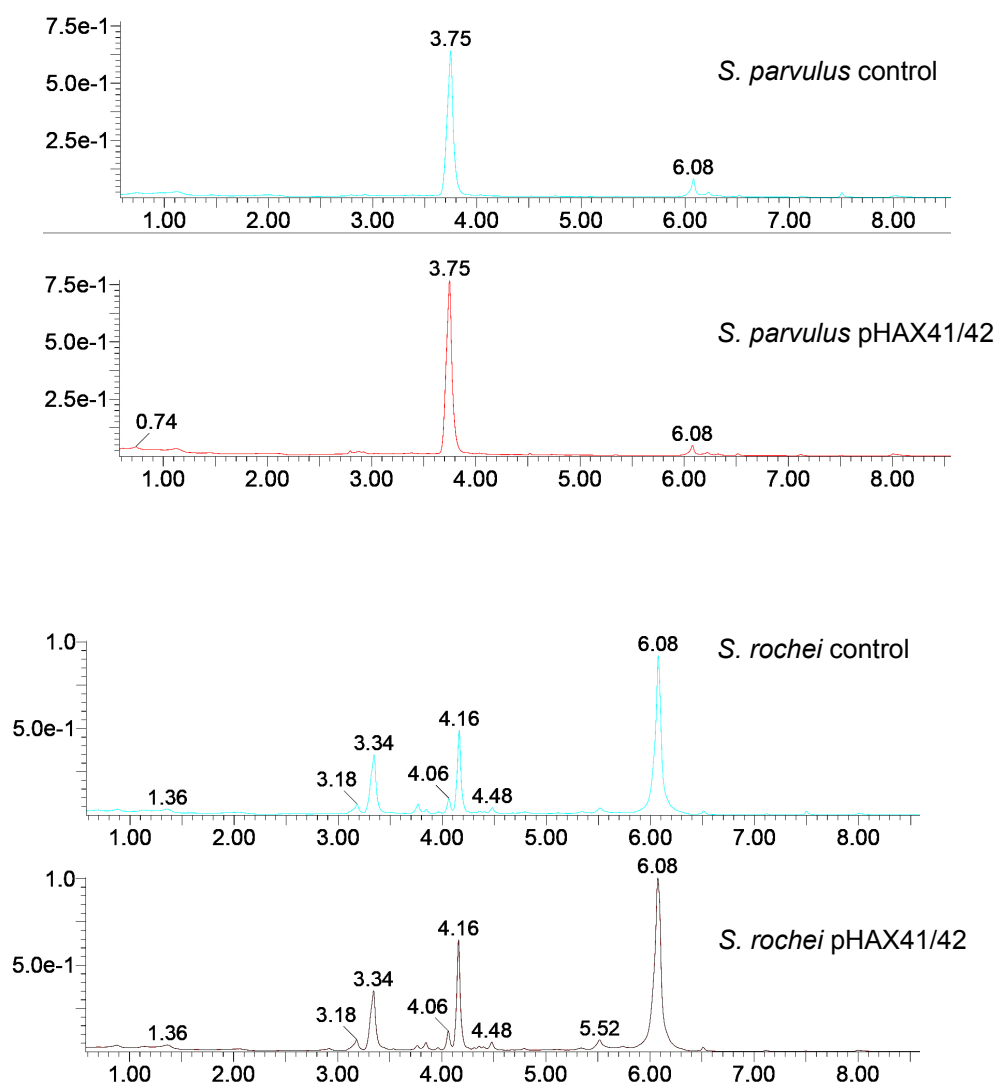

Figure S4: Chromatograms of *S. parvulus* and *S. rochei* grown in liquid YES+Xyl for 8 days.

Supplement: Supplementary file 4 [file Data_Sheet_4.PDF]

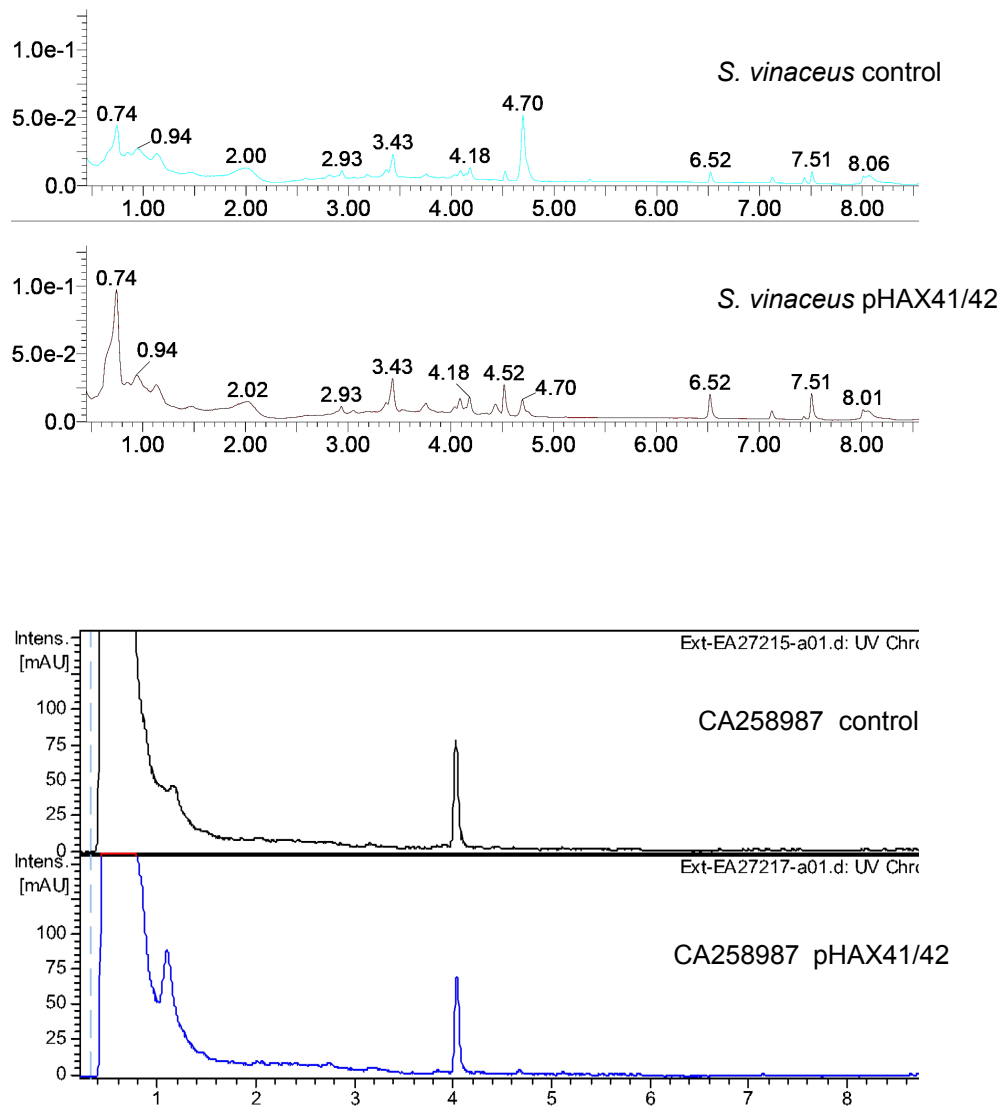

Figure S5: Chromatograms of *S. vinaceus* and *Streptomyces sp.* CA258987 grown in liquid YES+Xyl for 8 days.

Supplement: Supplementary file 5 [file Data_Sheet_5.PDF]
