# Supplementary material for: The XRE-DUF397 Protein Pair, Scr1 and Scr2, Acts as a Strong Positive Regulator of Antibiotic Production in Streptomyces
Source: Front Microbiol. 2018 Nov 16;9:2791. doi: 10.3389/fmicb.2018.02791 (PMC6262351; doi:10.3389/fmicb.2018.02791)
Supplement: Supplementary file 6 [file Data_Sheet_6.PDF]

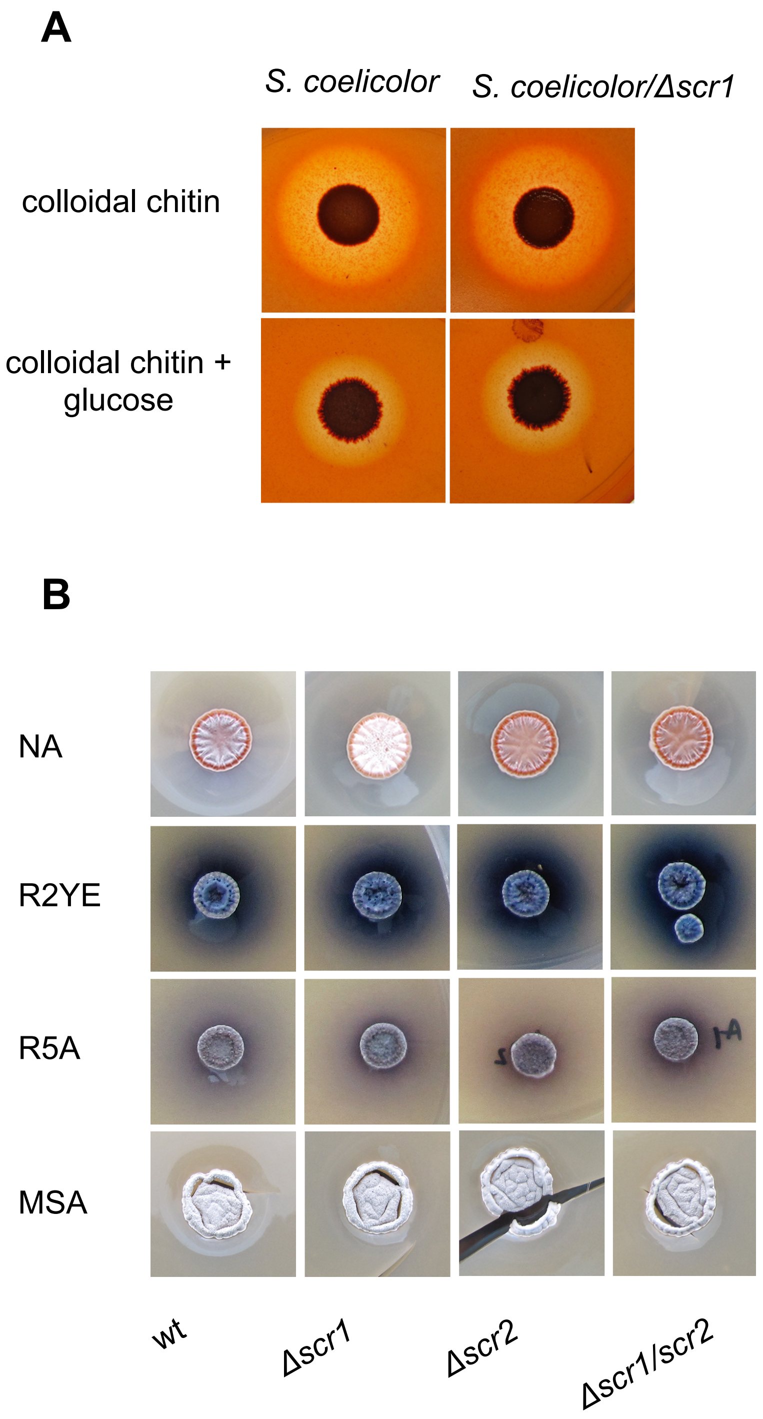

Figure S6: A) Chitinase production in *S. coelicolor* and in *S. coelicolor Δscr1* in ISP4+0,15 % colloidal chitin without or with 1 % glucose. B) Antibiotic production of *S. coelicolor* strains in the indicated solid media after 7 days of culture.
