# Supplementary material for: The XRE-DUF397 Protein Pair, Scr1 and Scr2, Acts as a Strong Positive Regulator of Antibiotic Production in Streptomyces
Source: Front Microbiol. 2018 Nov 16;9:2791. doi: 10.3389/fmicb.2018.02791 (PMC6262351; doi:10.3389/fmicb.2018.02791)
Supplement: Supplementary file 7 [file Data_Sheet_7.PDF]

Table S1: Origin of the 8 *Streptomyces* sp.

Strain	Taxonomy	Country	Sample type	Ecology
CA-240608	Streptomyces sp.	Central African Republic	Soil	Tropical humid forest
CA-243829	*Streptomyces* sp.	Central African Republic	Soil	Waterlogged forest
CA-135535	*Streptomyces* sp.	South Africa	Soil	Soil from Olive (*Olea africana*)
CA-248979	*Streptomyces* sp.	Georgia	Rhizosphere soil	Rhizosphere soil of *Neotorullaria eldarica*
CA-256884	*Streptomyces* sp.	Georgia	Rhizosphere soil	Rhizosphere soil of *Salix babilonica*
CA-258987	Streptomyces sp.	Georgia	Rhizosphere soil	Rhizosphere soil of *Digitalis ferruginea*
CA-259150	*Streptomyces* sp.	Georgia	Rhizosphere soil	Rhizosphere soil of *Astragalus stevenianus*
CA-243318	*Streptomyces* sp.	New Zealand	Soil	Canopy *Beilschmiedia tawa, Prumnopitys taxifolia, Melicytus* sp.
