# Supplementary material for: The XRE-DUF397 Protein Pair, Scr1 and Scr2, Acts as a Strong Positive Regulator of Antibiotic Production in Streptomyces
Source: Front Microbiol. 2018 Nov 16;9:2791. doi: 10.3389/fmicb.2018.02791 (PMC6262351; doi:10.3389/fmicb.2018.02791)
Supplement: Supplementary file 8 [file Data_Sheet_8.PDF]

Table S2: oligonucleotides used

Primers	Sequence (5'-3')	Function
LS-048	tttttcatATGAGTCACGCAGCCGGAGGTG	Forward oligonucleotide for amplifying *SCO4441*(*scr1*). The sequence recognized by NdeI is underlined.
LS-049	tttttctcgagGCGTGCGTACTCCTTCGCCAC	Reverse oligonucleotide for amplifying *scr1*. The sequence recognized by XhoI is underlined.
LS-050	tttttcatATGGCAATTCTTCAGGGCGCCC	Forward oligonucleotide for amplifying *SCO4442* (*scr2*). The sequence recognized by NdeI is underlined.
LS-051	tttttctcgagCTCCTTGACCGAGGTCACGAAG	Reverse oligonucleotide for amplifying *scr2*. The sequence recognized by XhoI is underlined.
LS-090	CACCTTGGTGGCAGTCTGCTCAGCAGCA GATACACAAAGTGGCAAGT**ATG**ATTCCG GGGATCCGTCGACC	Forward oligonucleotide to obtain the *scr1*mutation. The initiation codon is highlighted.
LS-091	GGCGAGTCTTCCAGTACATCGCAGCAGG GGGTAAGATGCCGCACCGC**TCA**TGTAGG CTGGAGCTGCTTC	Reverse oligonucleotide to obtain the mutant *scr1*. The termination codon is highlighted.
LS-092	GCGTGTACGCGGCGCATATCG	External forward oligonucleotide to check *scr1* mutation.
LS-093	CGTACGGAGCAGCCATTCACTC	External forward oligonucleotide to obtain *scr1* mutation.
LS 109	AACGTCATTGGCGTAAACCACACTCGCG ACTCGCCATCGGAGCAGAC**ATG**ATTCCG GGGATCCGTCGACC	Forward oligonucleotide to obtain the mutant *scr2*. The initiation codon is

		highlighted.
LS-110	GTCGAGAGGGCTCCGTCGGTGGTACCGGCGGGTGGCGCGTGGCGGCG**TTA**TGTAGGCTGGAGCTGCTTC	Reverse oligonucleotide to obtain the mutant *scr2*. The termination codon is highlighted.
LS-111	ACGGGAAGGCCAGCGAGTAGC	External forward oligonucleotide to check *SCO4442*.
LS-112	CGGGCGCCCTCTCGGCAAG	External reverse oligonucleotide to check *SCO4442* mutation.
